# Aromatase Deficiency due to a Novel Mutation in *CYP19A1* Gene

**DOI:** 10.4274/jcrpe.0011

**Published:** 2018-11-29

**Authors:** Edip Unal, Ruken Yıldırım, Funda Feryal Taş, Vasfiye Demir, Hüseyin Onay, Yusuf Kenan Haspolat

**Affiliations:** 1 Dicle University Faculty of Medicine, Department of Pediatric Endocrinology, Diyarbakır, Turkey; 2 Diyarbakır Children’s Hospital, Clinic of Pediatric Endocrinology, Diyarbakır, Turkey; 3 Kocaköy Family Health Center, Diyarbakır, Turkey; 4 Ege University Faculty of Medicine, Department of Medical Genetics, İzmir, Turkey

**Keywords:** Aromatase deficiency, CYP19A1 gene, maternal virilization, ambiguous genitalia

## Abstract

Aromatase deficiency is a rare autosomal recessive genetic disorder with an unknown incidence. Aromatase converts androgens into estrogen in the gonadal and extra-gonadal tissues. Aromatase deficiency causes ambiguous genitalia in the female fetus and maternal virilization (hirsutism, acne, cliteromegaly, deep voice) during pregnancy due to increased concentration of androgens. A 19 months old girl patient was assessed due to presence of ambiguous genitalia. There were findings of maternal virilization during pregnancy. The karyotype was 46,XX. Congenital adrenal hyperplasia was not considered since adrenocorticotropic hormone, cortisol, and 17-hydroxyprogesterone levels were within normal ranges. At age two months, follicle-stimulating hormone and total testosterone levels were elevated and estradiol level was low. Based on these findings, aromatase deficiency was suspected. A novel homozygous mutation IVS7-2A>G (c.744-2A>G) was identified in the *CYP19A1* gene. Pelvic ultrasound showed hypoplasic ovaries rather than large and cystic ovaries. We identified a novel mutation in the *CYP19A1* gene in a patient who presented with ambiguous genitalia and maternal virilization during pregnancy. Presence of large and cystic ovaries is not essential in aromatase deficiency.

**What is already known on this topic?**

Aromatase deficiency is an autosomal recessive genetic disorder that is rarely reported in the literature. Aromatase enzyme converts androgens into estrogen in many tissues. Aromatase deficiency causes ambiguous genitalia in the female fetus and maternal virilization during the pregnancy due to increased concentration of androgens. Ovaries are usually large and polycystic in girls with aromatase deficiency.

**What this study adds?**

We identified a novel mutation in the *CYP19A1* gene in a patient who presented with ambiguous genitalia and maternal virilization during pregnancy. In our patient, the ovaries were hypoplasic despite increased gonadotropin levels.

## Introduction

Aromatase is a member of the cytochrome P450 superfamily and encoded by the *CYP19A1* gene located on chromosome 15q21.1 (1). It is the key enzyme for estrogen biosynthesis in all vertebrates. *CYP19A1* gene and aromatase are expressed in numerous tissues including ovaries, testes, placenta, adipose tissue, skin and brain. Aromatase catalyzes the three precursors including androstenedione, testosterone and 16-α-hydroxy dehydroepiandrosterone sulfate (after conversion to 16-α-hydroxyandrostenedione) into estrone, estradiol and estriol, respectively (1,2,3). Aromatase deficiency leads to increased androgen levels both in the mother and the fetus. Aromatase deficiency causes specific signs of maternal virilization including cystic acne, hirsutism, cliteromegaly and deep voice while resulting in significant masculinization in the external genitalia of the female fetus (4).

In this study, we present a case with a novel homozygous IVS7-2A>G (c.744-2A>G) mutation in the *CYP19A1* gene causing significant virilization both in the mother and the female fetus.

## Case Report

The patient was born at term at another centre via spontaneous vaginal delivery with a birth weight of 3500 g. The parents were first-degree cousins. Ambiguous genitalia were recognized at birth. Signs of maternal virilization (hirsutism, acne, cliteromgaly, deep voice) were noted at approximately 20 weeks of gestation. The patient represented at age fifteen days. Congenital adrenal hyperplasia was not considered since adrenocorticotropic hormone, cortisol, and 17-hydroxyprogesterone levels were within normal ranges. The other parameters were as follows: follicle-stimulating hormone (FSH) 66 mIU/mL (0.24-14.2), luteinising hormone (LH) 9.7 mIU/mL (0.02-7.0), total testosterone 0.9 ng/mL (0.2-0.64), estradiol 5 pg/mL (<15). The karyotype was 46,XX and pelvic ultrasonography revealed the uterus dimensions as 5x8x13 mm (normal range 33.1±4.1 mm for uterus long axis), those of the right ovary as 5x3x3 mm (0.02 mL; normal range 0.2-0.9 mL), and those of the left ovary as 5x3x3 mm (0.02 mL; normal range 0.2-0.9 mL).

**Figure 1:**
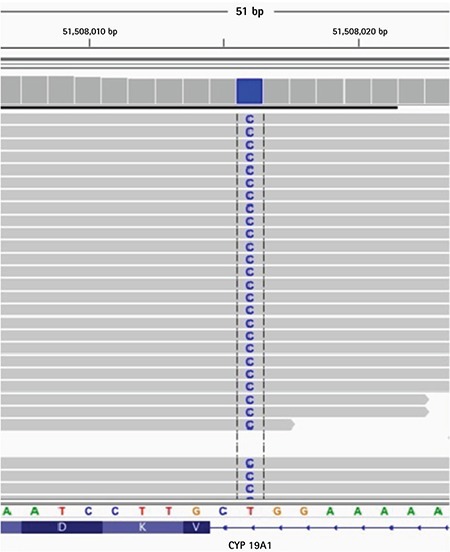
Homozygous mutation IVS7-2A>G (c.744-2A>G) in intron 7 of the *CYP19A1* gene

**Figure 2:**
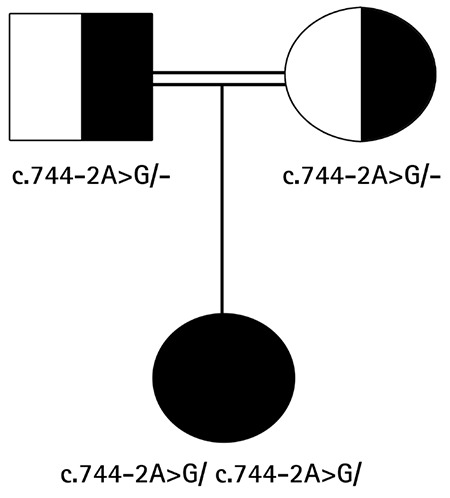
Pedigree of the patient’s family. Solid black symbols depict affected individuals; half-filled symbols represent heterozygous carriers

At presentation to our centre at 19 months of age she had ambiguous genitalia. The patient had a body weight of 11 kg [standard deviation (SD) score + 0.07] and a length of 82 cm (SD score + 0.48). Genital examination showed bilateral impalpable gonads, a penis-like phallus of 1.5 cm, single penoscrotal urethral opening, and labioscrotal fusion defect (Prader stage 4). Hormonal analyses were unremarkable except for a significantly elevated FSH level (Table 1). Aromatase deficiency was considered due to the presence of maternal virilization, detection of hypergonadotropic hypogonadism during mini-puberty and low estradiol levels despite elevated total testosterone levels. *CYP19A1* gene mutation analysis was performed by sequencing the coding exons and the exon-intron boundaries of the genes. Genomic DNA was isolated from peripheral blood cells with QIAGEN DNA Blood Midi Kit according to the manufacturer’s protocol. To amplify the exons of the *CYP19A1* gene, primers were used as listed in Table 2. Sequencing was performed with MiSeq V2 chemistry on a MiSeq instrument (Illumina California, USA) and the analysis was performed with IGV software. A novel homozygous [IVS7-2A>G (c.744-2A>G)] mutation was found in the *CYP19A1* gene (Figure 1). To our knowledge, this mutation has not been previously reported. The mutation was interpreted as a “disease-causing” mutation by the MutationTaster and Splice Site Finder modeling programs. The parents were heterozygous carriers for the same mutation (Figure 2).

Informed consent was obtained from the parents for publication of the case.

## Discussion

Aromatase deficiency is a rare disease caused by *CYP19A1* gene mutation and characterized by a decrease in estrogen synthesis. Aromatase deficiency is an autosomal recessive disorder and was first described by Shozu et al (5). To date, a total of 36 cases from various ethnic origins have been reported in the literature (1,2,6,7,8,9,10,11,12,13,14). In patients with aromatase deficiency, more than 30 distinct mutations have been identified in the *CYP19A1* gene, including missense, nonsense, small deletions and insertions, splice-site mutations, and one large intragenic deletion (1,2,6,7,8,9,10,11,12,13,14,15,16). Most of these mutations have been found to be located in exon 9 and 10 (9). The mutations identified in cases from Turkey have been reported in different exons (exon 5, 10, 11) (16,17,18). In our patient, the mutation was located in intron 7 of the *CYP19A1* gene.

Clinical characteristics of patients with aromatase deficiency vary depending on gender, age and enzymatic activity (1). Aromatase deficiency leads to an increase in intrauterine androgen concentration, thereby result in varying degrees of postnatal virilization in the external genitalia in girls and no change in the external genitalia in boys at birth. Our patient had a karyotype of 46,XX and was born with ambiguous genitalia (Prader stage 4). During infancy and childhood there are usually no symptoms of aromatase deficiency (particularly in boys) while some girl patients may present with abdominal symptoms of ovarian cysts because of mild changes in the hypothalamic-pituitary-gonadal axis due to lack of feedback regulation (3). Aromatase deficiency may lead to a number of clinical conditions in adolescent girls such as delayed puberty, hypergonadotropic hypogonadism, multicystic ovaries and primary amenorrhea in accordance with estrogen deficiency. Signs of virilization such as acne, hirsutism, and cliteromegaly in keeping with androgen excess may also be present (1,2,19,20). Estrogen deficiency, on the other hand, causes delayed epiphyseal closure, eunuchoid body habitus, osteopenia and osteoporosis that develop in both genders (21). A previous study reported a 27-year-old patient with bone pain and recurrent bone fractures secondary to minor trauma. The patient had open epiphyses and also developed lumbar osteoporosis. Aromatase deficiency was detected and the study concluded that estrogen has a key role in maintaining bone mineral density (17).

In most of the fetuses with aromatase deficiency, early (12 weeks) or late onset (up to 30 weeks) maternal virilization can be noted (20,22). The non-aromatized fetoplacental and maternal androgen precursors are converted to testosterone in the placenta and also in peripheral maternal tissues, thereby resulting in maternal virilization. After giving birth, the signs of virilization disappear gradually and the androgen levels return to normal (1). In our patient, the signs of maternal virilization (hirsutism, acne and deep voice) developed at approximately 20 weeks of gestation. Although hirsutism and acne resolved after birth, interestingly the deep voice persisted, which was consistent with the literature (13).

Both basal and GnRH-stimulated FSH levels have been shown to be higher in girls with aromatase deficiency during the first two years of life compared to normal subjects (50-75 and 200-255 mIU/mL, respectively). However, the estradiol and estrone levels tend to be remarkably low during this same period (22,23). Moreover, basal LH is often within normal limits or slightly elevated during infancy (5-10 mIU/mL). A previous study showed that in a girl with aromatase deficiency, the FSH and LH levels persistently increased and multicystic ovaries developed between the ages of three and four years (22). However, Belgorosky et al (23,24) reported that the basal FSH and LH levels in a girl with aromatase deficiency were found to be increased during mini-puberty and to show a dramatic decrease between two and five months. In our patient, gonadotropin (FSH, LH) levels were found to be elevated since birth.

In girls with aromatase deficiency, the ovaries are usually large and polycystic in every stage of life (newborn, childhood and puberty) due to the chronic stimulation by gonadotropins that cannot be suppressed owing to estrogen deficiency or androgen excess (1,2). In our patient, no cystic formation was observed in the ovaries despite high gonadotropin levels and also the ovarian volumes were below the age-matched limits. To date, hypoplasic ovaries have been reported in a total of five cases from three studies, the characteristics of which were similar to those of our patient (9,16,18).

Literature reviews indicate that there is little documentation on the effects of estrogen replacement to prevent estrogen deficiency in women with aromatase deficiency. Moreover, there is no consensus on the dosage and age of initiation of estrogen replacement therapy. On the other hand, data regarding early initiation of the treatment and the long-term follow-up of the patients are extremely rare. To our knowledge, there has been only one study investigating the effects of estrogen replacement therapy on longitudinal growth, bone age maturation, multicystic ovaries, bone density and regulation of the pituitary gonadotropin feedback in a girl with aromatase deficiency who was started on low-dose estrogen therapy at the age of 3.5 years and continued the therapy until the age of 15 years. The study revealed that estrogen is required for normal growth, pituitary-gonadal development and bone maturation not only in puberty but also in early childhood (3). In a review of treatment of aromatase deficiency, it was reported that estrogen replacement therapy can be initiated at as early as two years of age. The study also noted that this treatment should be initiated and sustained with the lowest dose of estrogen possible to prevent the development of ovarian cysts and to avoid early development of breasts and acceleration of bone age. The study suggested that oral conjugated estrogens (0.15 mg/day or every other day) or micronized estradiol (0.25 mg/day or every other day) can be used and the dose may be titrated to maintain the suppression of FSH and LH (4). In view of these findings, low-dose estrogen replacement therapy was planned for our patient at age two years for enhancement of the development of uterus and ovaries, normal growth, bone maturation and normalization of bone mineral density.

In conclusion, the case reported here presented with ambiguous genitalia and exisiting aromatase deficiency, findings which were due to a novel mutation in the *CYP19A1* gene. Presence of large and cystic ovaries is not essential in aromatase deficiency. On the contrary, the ovaries may be hypoplastic as in this case and a number of other previous reports (9,16,18). Aromatase deficiency should be kept in mind in patients with 46,XX karyotype presenting with ambiguous genitalia along with the signs of maternal virilization.

## Figures and Tables

**Table 1:**
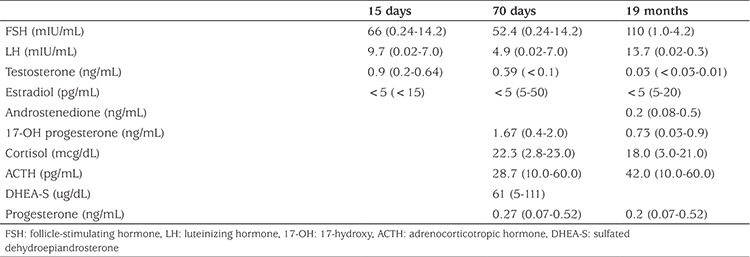
Hormone levels of the patient at different age time points

**Table 2:**
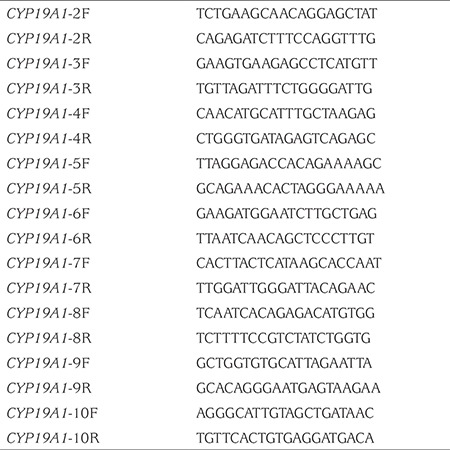
Primers used for sequencing the coding region of the *CYP19A1* gene
